# WISC-V Measurement Invariance According to Sex and Age: Advancing the Understanding of Intergroup Differences in Cognitive Performance

**DOI:** 10.3390/jintelligence11090180

**Published:** 2023-09-06

**Authors:** Marcela Rodríguez-Cancino, Andrés Concha-Salgado

**Affiliations:** Department of Psychology, Universidad de La Frontera, Temuco 4811322, Chile; andres.concha@ufrontera.cl

**Keywords:** Wechsler Intelligence Scale for Children, fifth edition, measurement invariance, sex, age, cognitive assessment, equivalence

## Abstract

This study sought to verify whether the constructs measured on the WISC-V are equivalent according to sex and age group in Chilean students to substantiate intergroup comparisons. For this, the measurement invariance of two variants of the five-factor intelligence model was explored with the ten primary subtests (hierarchical and oblique) using multigroup confirmatory factor analysis. Seven hundred and forty participants between 6 and 16 years of age from the Chilean standardization sample were assessed. The results show complete invariance according to sex, but incomplete according to the age group. The implications of these findings in both the professional area of psychology and future research are discussed.

## 1. Introduction

In order to use a test ethically and rigorously within a psychological assessment process, the quality of its psychometric properties must be explored and guaranteed (Muñiz et al. 2015). This refers, for example, to the reliability of its results, evidence of the validity of the interpretations that emerge from its scores, updated and representative normative data on the target population, and proven fairness, i.e., the absence of biased elements (in its items, materials, etc.) deleterious to the performance of a particular group (APA 2020; AERA et al. 2018; Muñiz et al. 2015; Muñiz and Fonseca-Pedrero 2019; Vinet et al. 2023).

Given the tremendous professional responsibility involved in test use for diagnosis, intervention, or treatment decision-making, verifying its fairness should be explored before large-scale implementation (Marín 2021; Vinet et al. 2023). At the psychometric level, some strategies that can test for fairness are measurement invariance analysis and differential item functioning (DIF). These techniques can identify if a measurement instrument is free of bias, endorsing the use of its scores in a defined context and population (Elosua 2003; Gómez-Benito et al. 2010; International Journal of Testing 2017; McGill et al. 2020; Muñiz and Fonseca-Pedrero 2019; Pedrero 2019).

### 1.1. Measurement Invariance

In psychology measurement, a test that is designed to measure a specific attribute should reveal differences between individuals only if those individuals actually differ in the attribute measured; if this is not met, it is possible to suspect the existence of a measurement bias which is equivalent to the systematic inaccuracy of the measurement (Millsap 2011).

In clinical or educational assessment, when the norms of a test have been developed for the general population, it is relevant to ask whether these norms can be applied in the same way in the assessment of individuals belonging to the sub-groups of a population, according to their sex, educational level, age, and ethnicity, among others. Given this, measurement invariance allows us to establish whether a test manages to generate a fair measure of the performance of people belonging to different sub-groups of the population, or in other words, whether it allows us to determine if the test works in an equivalent (invariant) way among the groups compared. When this is not demonstrated, it allows us to think about the need to have specific norms for the sub-groups of a population (Wicherts 2016).

Measurement invariance analysis can verify if the association between the items (or test scores) and the latent factors (or latent traits that are being measured) of the individuals do not depend on belonging to a particular group, i.e., whether it is invariant or stable among the groups being compared (Van de Schoot et al. 2012). Group membership should not alter the relationships between the observed and latent variables (Olivera-Aguilar et al. 2018). The invariant scores support the quality of the inferences made after comparing the sub-groups of a population on a test, guaranteeing that the differences in the scores are not related to characteristics inherent to the group being tested nor biases in the test, but rather to real variations in the measured construct (Chen et al. 2015; Elosua 2005; Leitgöb et al. 2023; Pauls et al. 2020; Pedrero 2019; Van de Schoot et al. 2012).

The factors that generate measurement bias in a test could be how the subtests are administered, cultural aspects of its items, and examinee-related problems such as familiarity with the content, language problems, and educational differences. They must be detected to ensure proper test use, since even Fluid Reasoning tests considered “culture-free” may exhibit measurement biases (Wicherts 2016).

According to Putnick and Bornstein (2016) and Dimitrov (2010), the levels of invariance are usually defined as (1) configural invariance (equivalence of the factorial structure), (2) metric invariance (*weak invariance*, equivalence of factor loadings), (3) scalar invariance (*strong invariance*, equivalence of the intercepts), and (4) residual invariance (*Strict invariance*, equivalence in the indicator residuals). Reaching the level of configural invariance demonstrates that in the compared groups, the latent factors are specified by the same manifested variables (Elosua 2005). Then, the equivalence of the factor loadings that reflect the relevance of these indicators of the factor they define would be shown when the level of metric invariance is reached. 

Subsequently, researchers should demonstrate scalar invariance, which means that the mean differences in the latent construct capture all the mean differences in the shared variance of the items (Putnick and Bornstein 2016). Therefore, the equivalence of the intercepts would imply that the compared groups have the same starting point in the measured variable. For example, if the scores on a vocabulary subtest of an intelligence test are compared between boys and girls, the equivalence of the intercepts would mean that both groups have the same mean score on this subtest conditional on the ability, although their means differ across other variables. The equivalence of the intercepts is essential because, if not fulfilled, the differences in the means of the measured variables between the groups cannot be solely attributed to group differences in the measured construct. In other words, if the equivalence of the intercepts is violated, the scores of the two groups cannot be comparable, and it cannot be valid to conclude the differences in the underlying construct (Van de Schoot et al. 2012). 

Finally, residual invariance means that the sum of specific variance and error variance is similar across groups; namely, if it is possible to demonstrate the most demanding level of measurement invariance, it means that the residuals between the different groups are equivalent, which suggests that the measurement errors are similar between the groups (Putnick and Bornstein 2016). Although demonstrating residual invariance is essential for determining full factorial invariance, this step is not a prerequisite for comparing mean differences because the residuals are not part of the latent factor (Vandenberg and Lance 2000).

Establishing the measurement invariance of a test is an indispensable requirement within a practical or professional setting (Meredith 1993) and the construction of norms when a test is used within multicultural contexts (Wicherts 2016). Likewise, in the scientific field, comparisons between groups should only be made once it has been proven that the constructs measured are understood in the same way among the groups compared, which can be explored and guaranteed with measurement invariance analyses (Chen et al. 2015). 

In the field of intelligence assessment, testing for measurement invariance is an excellent way to empirically explore whether the subtests or items of a test that measures cognitive abilities function in the same way across different sub-groups of a population to support the use of its norms (Wicherts 2016). Several studies on measurement invariance with the most well-known cognitive assessment instruments (WISC-R, WISC-IV, WAIS-III, WAIS-IV, JAT, RAKIT, KAIT, WJ IV COG) have shown varying results depending on the ethnicity, sex or age of the participants, showing that some of them pass tests of invariance (Chen et al. 2015; Dolan 2000; Tommasi et al. 2015), some fail (Dolan et al. 2004; Wicherts et al. 2004; Wicherts and Dolan 2010), and others partially achieve it (Abad et al. 2016; Bulut et al. 2021; Chen and Zhu 2012; Dolan et al. 2006; Immekus and Maller 2010). According to Wicherts (2016), it is disturbing to see that the tests commonly used in clinical or educational practice do not pass invariance tests, since they would be demonstrating that the scores obtained are not only due to differences in the latent cognitive abilities being measured, which should encourage the creation of specific sub-groups of a population, or bias corrections in the test items or subtests.

Another test with which invariance testing studies have been carried out in various parts of the world is the Wechsler Intelligence Scale for Children in its fifth edition (WISC-V), considering variables such as sex, ethnic group or age groups, among others (Chen et al. 2015; Dombrowski et al. 2021; Pauls et al. 2020; Reynolds and Keith 2017; Scheiber 2016). This scale evaluates cognitive functioning in childhood and adolescence by obtaining the total intelligence quotient (FSIQ), five primary indexes (Verbal Comprehension, Visual Spatial, Fluid Reasoning, Working Memory, Processing Speed), and five secondary indexes (Quantitative Reasoning, Auditory Working Memory, Nonverbal, General Ability, and Cognitive Competence), and currently includes of the evaluation of intelligence as the “gold standard,” used worldwide and adapted to various cultural realities (Chen et al. 2015; Coronel et al. 2006; Flanagan and Alfonso 2017; Giofrè et al. 2022; Niileksela and Reynolds 2019; Rosas et al. 2022; Wechsler 2014; Weiss et al. 2019).

WISC-V integrates the most recent contributions of the CHC (Cattell Horn Carroll) Theory of Intelligence, as well as evidence emanating from research in developmental psychology, cognitive neuroscience, and working memory measurement models (Canivez and Watkins 2016; Forns and Amador 2017; Wechsler 2014).

The CHC Hierarchical Model of Intelligence has been recognized as the first consensus, comprehensive, and empirically validated taxonomy of cognitive abilities (Brenlla 2013; Flanagan and Dixon 2014; McGrew et al. 2023). Theoretically underpins the interpretations of the results from the Wechsler scales (WAIS-IV, WISC-IV, and WISC-V in the United States), as well as other recognized test of wich the objectives are to measure the construct of intelligence (Brenlla 2013; Canivez et al. 2016). 

Similar to the CHC intelligence model, the conceptualization of intelligence and the factor structure of WISC-V considers a hierarchical structure that includes a general intelligence factor, five *broad level* primary factors, which in turn, contain more specific cognitive skills (*narrow level*), and measure skills of fluid intelligence (*Gf*), crystallized intelligence (*Gc*), working memory (*Gsm*), Visual Processing (*Gv*); Auditory Processing (*Ga*), Processing Speed (*Gs*), among others (Brenlla 2013; Kaufman et al. 2016; McGrew et al. 2023; Flanagan and Dixon 2014).

Van de Vijver et al. (2019) and Pauls et al. (2020) refer to the indispensability of investigating the factorial invariance of the WISC to verify the universality of the intelligence construct that supports the interpretation of the scores of this instrument., i.e., to confirm that the same theoretical structure is suitably adjusted for the different sub-groups being compared. 

A series of studies (shown in Table 1) have tested invariance of the WISC-V according to sociodemographic variables, finding mixed results. In some cultural realities, strict levels of invariance are reached in terms of participants’ sex, age, or ethnic group. In contrast, in others, its achievement is partial, considering the same grouping variables. This reveals an inconclusive panorama regarding the equivalence of the intelligence construct measured by this scale in different population sub-groups. Table 1 summarizes the main findings reported in scientific literature on invariance studies with the most up-to-date version of the WISC.

### 1.2. WISC-V Invariance According to Sex

As noted in Table 1, evidence of configural, metric, scalar, and residual invariance has been found according to the sex of those being evaluated in the standardization sample in the United States (Chen et al. 2015) and Taiwan (Chen et al. 2020). Also, evidence of configural, metric, and scalar invariance has been reported according to sex in the Afro-American, Hispanic, and Caucasian populations (Scheiber 2016) and clinical samples (Dombrowski et al. 2021) that endorse the interpretative strategy and performance comparisons among these groups. 

Pauls et al. (2020) in Germany found partial scalar invariance according to the participants’ sex, noting that four of the fifteen subtests belonging to the cognitive domains of Verbal Comprehension, Fluid Reasoning, and Processing Speed were not stable between boys and girls. In addition, Smith and Graves (2021) found a level of partial scalar invariance according to sex in a sample of Afro-American children, demonstrating inequality in the subtests of Similarities (Verbal Comprehension) and Coding (Processing Speed). These results suggest that it is impossible to compare these groups and question the quality of the interpretations made from the performance of those evaluated with a possible presence of measurement bias (Meredith 1993).

### 1.3. WISC-V Invariance According to Age Group

Unlike the variable sex, measurement invariance of the WISC-V based on the age group has been less explored, emphasizing evidence of strict invariance only within the standardization sample in the United States (Reynolds and Keith 2017) and Taiwan (Chen et al. 2020). For their part, Dombrowski et al. (2021) only reached partial scalar invariance with the age group (6–8; 9–11; 12–14; 15–16), identifying imbalances in the subtests that comprise the cognitive domain of Fluid Reasoning. This lack of invariance on the most demanding levels indicates that the meaning of the measured constructs differs between the compared groups and that the scores are possibly affected by some measurement bias that must be examined at a greater depth (Vandenberg and Lance 2000). 

### 1.4. WISC-V Invariance Studies in Chile

In Chile, the WISC-V has recently been standardized. It has psychometric evidence for its reliability, internal structure validity, its relation with other variables, and updated normative data in a broad sample of children and adolescents (Rosas et al. 2022). Regarding exploring fairness, only the study of Rodríguez-Cancino et al. (2021) has been conducted in Chile in a sample of 480 schoolchildren according to their origin (urban *n* = 320; rural *n* = 160). This study tested the invariance of the hierarchical model that includes the ten primary subtests grouped within five indexes, reaching the level of configural and partial metric invariance, in which nine of the ten subtests are invariant among the compared groups, except for the Similarities subtest that belongs to the Verbal Comprehension index. No other studies have explored measurement invariance according to the gender or age group in this country, neither with the current nor previous version of WISC.

### 1.5. Performance Comparisons with WISC-V According to Sex and Age

Concerning the variable sex, a study conducted with the standardization sample in the United States of WISC-V found higher scores for the indexes of Working Memory, Processing Speed, Nonverbal, Cognitive Competence, and FSIQ in girls. By contrast, boys only surpassed them on the Quantitative Reasoning index (Kaufman et al. 2016). Similar results were found in Spain (Hernández et al. 2017) and France (Grégoire 2020), where there was agreement that girls perform significantly superior to boys, particularly for the Processing Speed Index. 

A similar trend is observed in Chile, given that in the comparisons of the performance on the WISC-V according to sex, it is the girls who achieve higher scores than the boys, specifically in the domain of Processing Speed (Garolera and Navarro 2020; Rosas et al. 2022).

Among other studies that have explored the differences in the performance on the WISC according to the sex of those evaluated, it is relevant to emphasize the findings of Giofrè et al. (2022). The authors carried out a meta-analysis reviewing 79 studies conducted using different versions of the Wechsler scales for children (WISC, WISC-R, WISC-III, WISC-IV, and WISC-V). They found that, throughout the versions, the boys tend to present better performances in visual spatial and crystallized intelligence tasks. In contrast, girls demonstrate significantly superior performance in processing speed tasks, in addition to showing that for the older versions of WISC, more significant differences were observed attributable to sex than for the most recent versions. 

These findings are consistent with those reported by Grégoire (2020) in France, who analyzed the differences by sex in the performance on different versions of the WISC (WISC-R, WISC-III, WISC-IV, and WISC-V), with the data from the standardization samples demonstrating that the differences in performance between girls and boys have gradually been reduced through the versions of the scale, and that on the WISC-V, they disappear for FSIQ and in four of the five primary indexes. On the WISC-V, this author found better performance in the Figure Weights and Arithmetic subtests in favor of boys, and significantly higher performance of girls for Coding, Symbol Search, Cancellation, Comprehension, and Picture Span.

With other standardized instruments, a study by Bulut et al. (2021) found similar results indicating that girls perform better than boys in processing speed; however, boys surpass girls in the cognitive skills of working memory, visual spatial skills, and crystallized intelligence. According to these authors, the differences attributable to sex in intelligence vary between age groups and over time due to the inequality in the maturation rates between boys and girls, noting that younger girls surpass boys’ performance. This trend is inverted as they grow.

Regarding age groups, scientific literature has evidenced changes in cognitive ability development through childhood and adolescence (Ardila 2011; Sattler 2003). During childhood, intelligence tends to develop rapidly, with significant cognitive advancements occurring within various domains, such as language, memory capacity, problem-solving, and reasoning. As children transition into adolescence, their intelligence continues to evolve, although at a slower pace compared to childhood, and their cognitive abilities become more refined (Rosas et al. 2019). Adolescents typically experience enhanced reasoning skills, increased ability for abstract thinking, and improved executive functions such as planning, decision-making, and self-regulation (Center on the Developing Child at Harvard University 2011; Diamond 2013; Rosselli et al. 2010).

Because norms have been created according to age, no empirical studies have compared the performance of age groups on the WISC-V. However, there are studies of measurement invariance according to age, as mentioned previously.

As invariance testing is a mechanism to validate performance comparisons between groups, it is worth asking if the findings of differences in the performance on the WISC-V between boys and girls in Chile, and according to their age group, are possibly explained by real variations in the latent factor or by belonging to one of the compared groups. If explained by the latter, this will reflect the presence of measurement bias that would impede comparison and could reasonably make doubt the quality of the interpretations of the results.

On the other hand, determining the existence of biases in the measurement with WISC-V according to variables like sex or age group is especially critical in Chile, since no studies have produced evidence on these aspects. This instrument must be applied on a mandatory basis in the national school system to define the delivery of specialized support and establish the diagnosis of an intellectual disability (Ministerio Educación Chile 2009). Hence, it must have psychometric properties guaranteed for these purposes.

### 1.6. The Present Study

Having as a target the study of biases in the tests, the present study endeavors to answer international directives on the ethical use of instruments through the review of possible sources of fairness or bias of the WISC-V, to contribute to the accomplishment of fair psychological assessments and the incorporation of good practices in psychodiagnostics.

From the prepared problem and on a general level, the present study describes evidence of fairness on the WISC-V in measuring cognitive functioning among the Chilean child-youth population according to sex and age group. 

**Research question:** Are the constructs measured using WISC-V equivalent according to the sex and age group of Chilean children and adolescents?

**Hypothesis 1.**  *The factorial model of the WISC-V is invariant according to sex.*

**Hypothesis 2.**  *The factorial model of the WISC-V is invariant according to the participants’ age group.*

## 2. Materials and Methods

### 2.1. Participants

The sample comprised 740 children and adolescents between 6 and 16 years of age, stratified according to the school’s funding scheme (Municipal = 33.6%, Subsidized Private = 34.9%, and Private = 31.5%). The data were collected through the WISC-V Standardization Project in Chile (Rosas et al. 2022), conducted by a research team at the Centro de Desarrollo de Tecnologías de Inclusión de la Pontificia Universidad Católica de Chile (CEDETi-UC). 

The participants, from seven geographic zones across the country, were selected through non-probability purposive sampling, with the following inclusion criteria: (a) between 6 years and 16 years of age, and (b) not recently assessed with a similar scale. The exclusion criteria were a clinical diagnosis and/or permanent or temporary special education needs. Table 2 contains the complete information on the frequencies by sex and age group. The definition of the age groups for this study followed the previous evidence in studies that performed similar analyses, such as that by Chen et al. (2020) and the one by Dombrowski et al. (2021).

### 2.2. Instruments

#### Wechsler Intelligence Scale for Children, Fifth Edition (WISC-V)

This individually administered clinical instrument was developed to assess intellectual abilities in children and adolescents between 6 and 16 years of age (Rosas and Pizarro 2018; Wechsler 2014). The Chilean version of the WISC-V was standardized using data from 754 children and adolescents, stratified by socioeconomic level, and from 7 of the 15 regions of the country (Rosas et al. 2022). Regarding internal structure validity, the correlation matrix between the subtests and indexes in the Chilean sample behaved as theoretically expected (higher correlations between subtests belonging to the same cognitive domain). Also, the confirmatory factor analysis for the hierarchical model that includes ten primary subtests showed an excellent fit, where *χ*^2^(30) = 52.245, *p* < 0.001, *χ*^2^/*df* = 1.7, RMSEA = 0.031, CFI = 0.99, TLI = 0.99. The scale includes 15 subtests, of which 10 are primary and 5 complementary, organized within five cognitive domains, as illustrated in Table 3. Information about the reliability coefficients of the subtests and indexes for the Chilean sample is also included.

The Chilean version of the WISC-V obtains a Full Scale Intelligence Quotient (FSIQ) indicator after the administration of 7 primary subtests, 5 primary indexes, applying ten primary subtests and 5 secondary indexes, all standardized scores expressed on a point scale (M = 10, SD = 03) for the subtests and as composite scores (*M* = 100, *SD* = 15) for the indexes and FSIQ (Kaufman et al. 2016; Rosas and Pizarro 2018; Weiss et al. 2019).

### 2.3. Procedure

The data were collected by the WISC-V Standardization Project in Chile (Rosas et al. 2022), administering the instrument only to the children or adolescents who voluntarily agreed to participate (demonstrated by the signature of an informed assent) and whose parents authorized it by signing an informed consent form.

Each child was evaluated individually at their school and during the school day. The scale administration lasted between 60 and 90 min, the variation of which depended on the characteristics of each child evaluated (Rosas et al. 2022).

In terms of ethical safeguards, all the procedures carried out in the Standardization Project were approved by the Scientific Ethics Committee of the Pontificia Universidad Católica de Chile. The consent documents included explanations about the project’s aims, the instrument’s administration, and the right to withdraw from participating, at any time, without entailing any disadvantage. These documents specified safeguarding confidentiality and data use, indicating that they would be used anonymously and only for scientific and academic purposes.

To use the data in this study, authorization was sought from CEDETi-UC, the institution that owns the rights to the WISC-V in Chile and endorsed this study.

### 2.4. Data Analyses

**Figure 1:**
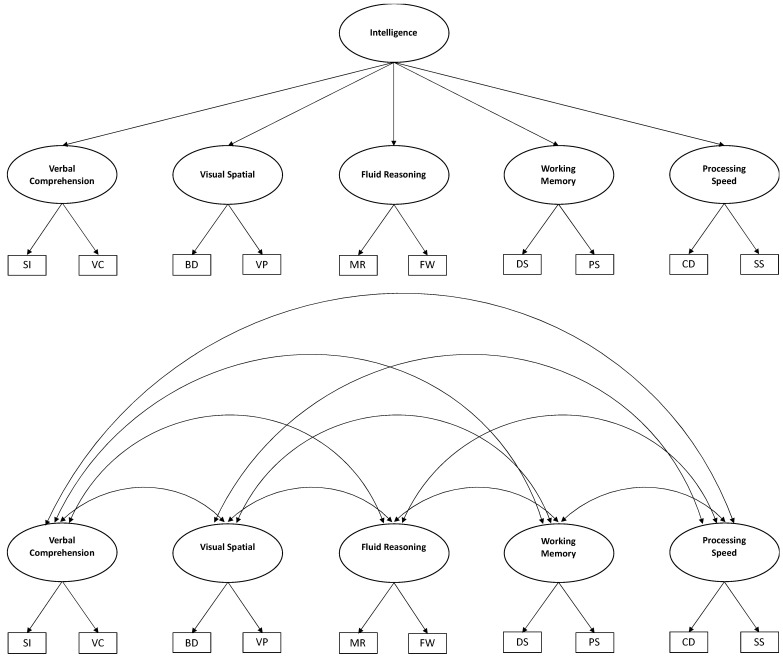
Hierarchical and oblique models of intelligence. *Note*: Figure created by the authors.

For the analyses of invariance, the hierarchical five-factor model proposed by Wechsler (2014) for the American standardization sample was chosen, which also has suitable levels of psychometric fit with the Chilean standardization sample (see Figure 1), made up of 10 primary subtests, 5 indexes and a second-order general intelligence factor (Rosas et al. 2022). Following the findings of several similar investigations (Dombrowski et al. 2021; Reynolds and Keith 2017; Smith and Graves 2021), an alternative first-order model was also tested that includes 5 primary indexes (oblique factors) and 10 primary subtests (indicators), corresponding to the structure of the first-level WISC-V constructs (see Figure 1).

As a preliminary step in the factorial analysis of invariance, the baseline factorial models were tested for the entire sample (*n* = 740) and separately for each group according to sex and age group through a first and second-order confirmatory factor analysis (CFA). It should be noted that the 11 age groups, from 6 to 16 years old, were clustered following the Chen et al. (2020) and Dombrowski et al. (2021) strategy, namely: 6–8, 9–11, 12–14, and 15–16. According to the guidelines of Putnick and Bornstein (2016) and Dimitrov (2010), different levels of nested models included more restrictions at each level successively, simultaneously for the groups through the multigroup confirmatory factor analysis (MGCFA).

Along the same lines as Dombrowski et al. (2021), a series of progressive restrictions were applied to the models to determine their equivalence based on sex (boy/girl) and age group. First, the level of configural invariance was explored by verifying whether the structure of the WISC-V was stable between groups without applying restrictions to the parameters of the models. After this, the factor loadings in each group were restricted, except for the one in the reference group. Then, the fit indexes were compared to those of the configural level to test the metric invariance (or weak) level. Later, restrictions were applied to the intercepts to determine the level of scalar invariance (or strong), and the fit was compared with the level of metric invariance. Finally, to test the level of residual invariance (strict), i.e., to assess if the residuals of the indicators are equivalent between the groups, they were restricted, and then their fit was compared to the level of scalar invariance (Dimitrov 2010; Putnick and Bornstein 2016). In the case of the hierarchical model, the first order was tested, and then the second order in each step of the progressive levels of invariance (Dimitrov 2010).

The analyses were performed with the maximum likelihood, using a robust standard error (MLR) estimator for both the CFA and the MGCFA. As indicators to establish the fit of the factorial models, the root mean square error of approximation (RMSEA), comparative fit index (CFI), and the Tucker-Lewis index (TLI) were considered. This way, the presence of an optimal fit was considered when the CFI and TLI ≥ 0.95, and RMSEA < 0.05, and a reasonable fit when the CFI and TLI ≥ 0.90, and RMSEA < 0.08 (Browne and Cudeck 1992; Hu and Bentler 1999).

The criteria to establish invariance in the comparison of the models (metric versus configural, scalar versus metric) were: variations ≥ 0.010 in CFI, ≥0.015 in RMSEA, or ≥0.030 in SRMR indicate the absence of metric invariance and a difference ≥ 0.010 in CFI, ≥0.015 in RMSEA or ≥0.010 in SRMR suggest the absence of scalar and residual invariance (Chen 2007; Chen et al. 2020; Cheung and Rensvold 2002). All data analyses were carried out with the standardized scores of the subtests using Mplus 8.5 software.

In order to determine whether there were differences based on sex on the 10 WISC-V sub-test scores and the 5 indexes, two one-way MANOVAs were conducted. Also, a one-way ANOVA was performed to compare the FSIQ between boys and girls. A partial eta squared (*η*^2^*_p_*) was used to study effect size considering the Cohen (1988) rule (0.01, small; 0.06, medium; 0.14, large).

## 3. Results

### 3.1. Descriptions of WISC-V Scores According to Sex

At the subtest level, and considering a mean of 10 and a standard deviation of 3, both boys and girls performed slightly higher than 10 in BD, SI, MR, DS, VC, FW, VP, and PS. In CD and SS, the boys were below 10, and girls above this theoretical average. Regarding skewness and kurtosis, most values were less than 0.5 in absolute value, indicating a general trend toward symmetric and mesokurtic distributions, consistent with the normal transformation of the scores. In the VCI, VSI, FRI, WMI, and FSIQ indexes, boys and girls obtained averages slightly above 100 points, and only males were below that average in PSI. The trend in the descriptive measures of shape also accounts for symmetric and mesokurtic distributions.

Box’s test of equality of covariances matrices and Levene’s test of equality of error variances were not significant; therefore, the assumptions of the MANOVA were demonstrated. As seen in Table 4, there were significant differences in favor of boys for DS: *F*(1, 707) = 4.495, *p* = 0.034; *η*^2^*_p_* = 0.006 (small). On the contrary, significantly higher means were observed in girls for CD: *F*(1, 707) = 21.994, *p* < 0.001, *η*^2^*_p_* = 0.030 (small) and SS, *F*(1, 707) = 6.929, *p* = 0.009, *η*^2^*_p_* = 0.010 (small). Regarding the indexes, only for PSI did girls outperformed boys, *F*(1, 707) = 19.244, *p* < 0.001, *η*^2^*_p_* = 0.026 (small). All other comparisons of the primary sub-tests, indexes, and the FSIQ, showed no significant effects.

### 3.2. Analysis of Invariance According to Sex and Age Group

#### 3.2.1. Baseline Models

The results of the CFA for the total sample and the groups separately show suitable fit indexes, both for the second-order five-factor model and for the first-order five-factor model, according to the sex and age of the participants (see Table 5).

#### 3.2.2. Configural Invariance

Evidence of equivalence was found for the factor structure of the hierarchical five-factor model regarding the sex (see Table 6) and age groups (see Table 7). Also, for the five-factor oblique model in the two variables (see Table 8 and Table 9).

#### 3.2.3. Metric Invariance

In the hierarchical five-factor model, the equivalence of factor loadings for sex was demonstrated both concerning first-order and second-order indicators as well as in the oblique five-factor model (see Table 6 and Table 8).

**Figure 2:**
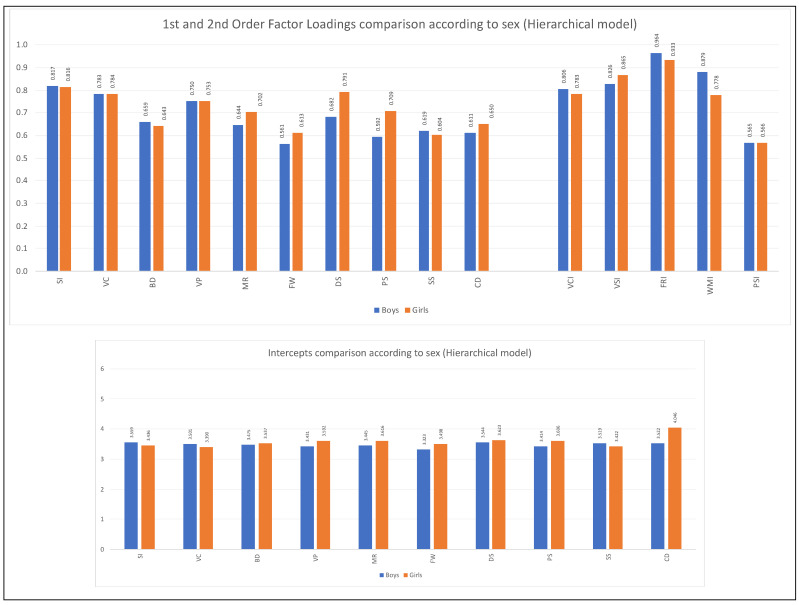
First and second-order factor loadings and intercepts comparisons according to sex **(hierarchical model).** *Note*: Figure created by the authors.

**Figure 3:**
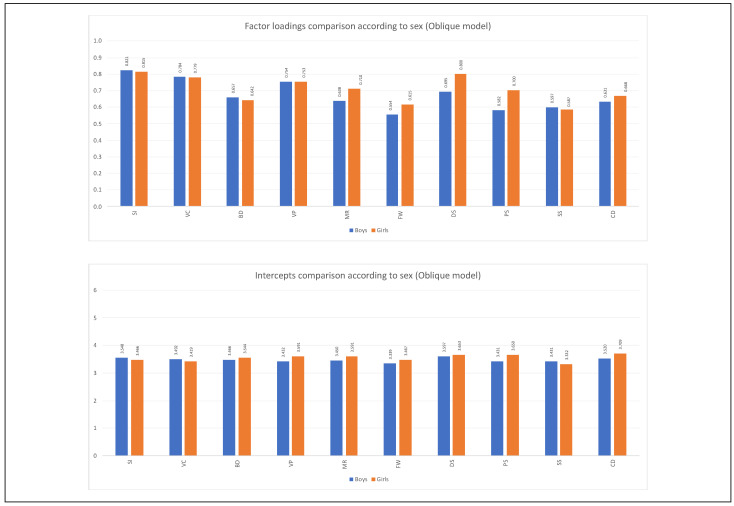
Factor loadings and intercepts comparisons according to sex **(oblique model).** *Note*: Figure created by the authors.

**Figure 4:**
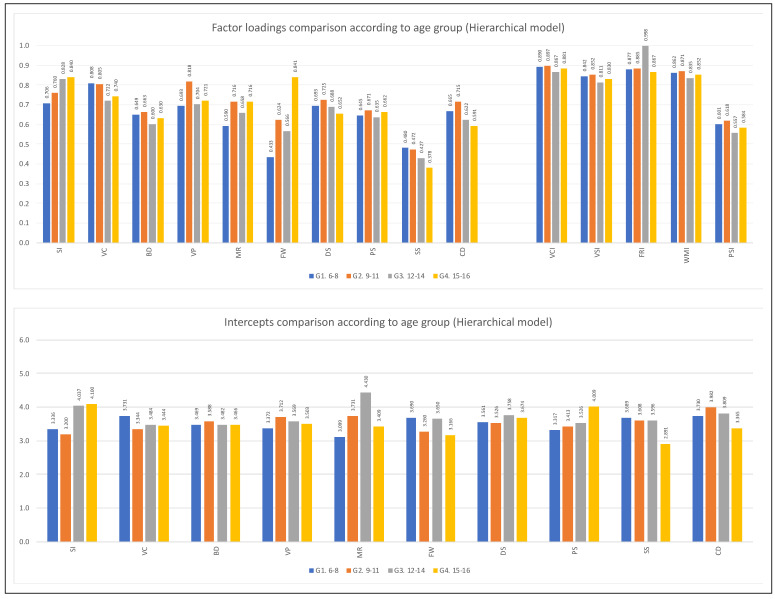
First and second-order factor loading and intercept comparisons according to age group (**hierarchical model**). *Note*: Figure created by the authors.

**Figure 5:**
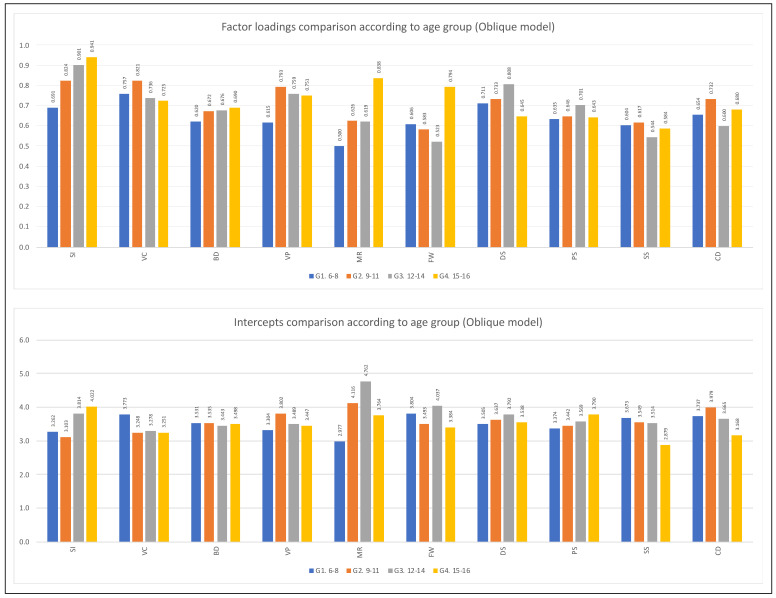
Factor loading and intercept comparisons according to age group (**oblique model**). *Note*: Figure created by the authors.

For the variable age group, only partial metric invariance was reached for the first-order indicators of the hierarchical five-factor model (see Table 7). The same occurred in the oblique five-factor model (see Table 9). According to the analyses reviewed, the most remarkable differences in the factorial weights are found in the subtests of the cognitive domain of Fluid Reasoning (Matrix Reasoning and Figure Weights) in the two factorial models tested. Figure 2, Figure 3, Figure 4 and Figure 5 show the comparison of the factor loadings for each model according to sex and age group.

#### 3.2.4. Scalar Invariance

Concerning the equivalence of the intercepts of the indicators according to the variable sex, the level of scalar invariance was reached in the second-order five-factor model. It should be emphasized that although the Δ*CFI* value slightly exceeds what is permitted, it was decided to continue with the analysis since the other two indicators were fulfilled perfectly (see Table 6). In the oblique five-factor model, the equivalence of the intercepts based on sex is verified (see Table 8). For the age group, the scalar invariance level was not reached (see Table 7 and Table 9). Figure 2, Figure 3, Figure 4 and Figure 5 show the comparison of the intercepts for each model according to sex and age group.

#### 3.2.5. Residual Invariance

Finally, the equivalence in the residuals of the indicators was reached only according to sex in both the hierarchical second-order five-factor model and the oblique five-factor model (see Table 6 and Table 8).

The summary of the reached invariance levels for the two models according to the two comparison variables can be seen in Table 10.

## 4. Discussion 

According to international guidelines, the verification of a test’s psychometric properties guarantees that its use is fair and relevant to a specific population (AERA et al. 2018). Professionally, having this information is especially helpful when an evaluator must select the instruments to use as part of a psychological evaluation, taking into account the evidence around the equivalence of the measurement or the information of the normative data. This allows to determine if the test is appropriate according to the sociodemographic characteristics of the person being evaluated (APA 2020). On the other hand, and in the scope of the study, exploring the measurement invariance would make it possible to clarify if the scores that people obtain on a test genuinely reflect the level of the evaluated construct, or if this could depend on belonging to a particular group of a population, a situation in which its use for making intergroup performance comparisons would be questionable (Chen et al. 2015; Elosua 2005). Thus, the evidence offered by the exploration of measurement invariance is valuable and necessary in psychology, both in professional practice and research. 

The present study sought to generate evidence about the fairness of the WISC-V, considering sociodemographic variables (sex and age group) in the measurement of the cognitive functioning of the Chilean child youth population to establish whether the constructs measured on the scale are equivalent within the sub-groups evaluated.

### 4.1. Invariance According to Sex

When taking the variable sex into account, the results show that in both the hierarchical and oblique models, the latent constructs are made up of the same manifested variables for boys and girls (configural invariance), i.e., there is equivalence in the form of measurement models, noting that the constructs of the WISC-V are measured with the same number of factors and the same number of indicators within each factor. Furthermore, in the following level of invariance (metric), equivalence was found for boys and girls in the factor loadings of each subtest, in the factor to which they belonged, establishing that in the two measurement models tested, the manifested variables are also predicted by the latent variables. These results are consistent with studies in other cultural realities, such as the United States (Chen et al. 2015; Dombrowski et al. 2021; Scheiber 2016), Taiwan (Chen et al. 2020), or Germany (Pauls et al. 2020).

Scalar invariance was also reached according to the sex of the participants in the oblique model, reflecting that boys and girls have the same starting point on the measurement scale of the subtests, i.e., they have the same subtests means. This finding demonstrates the absence of bias, and allows for the comparison of the results of these groups, since it is feasible to suppose that with the differences in the performance means in the measured variables, these would be attributable to fundamental differences in the latent construct. Moreover, these results are consistent with Dombrowski et al. (2021), who demonstrated scalar invariance according to sex using the oblique model.

The last level of measurement invariance tested in the present study (residual invariance) was also achieved according to the sex of the participants within the Chilean sample, similar to Chen et al. (2015) in the United States and Chen et al. (2020) in Taiwan, demonstrating equality among the residuals of the indicators in both models tested. If the residuals are invariant, then the variance, distribution, and correlation of the residuals are similar across all groups. This result reflects that sex differences in children’s performance on WISC-V are real differences in the constructs measured and cannot be attributed to measurement errors. Finally, it should be noted that the AIC indicator suggests that the higher-order model does not differ from the correlated model.

Thus, the results endorse the professional use of the interpretative and analytical strategy of the scores proposed by the WISC-V for Chilean boys and girls, i.e., the score calculation derives from grouping the 10 subtests that comprise the 5 primary indexes. In terms of research, the analysis of the differences in performance on the WISC-V must be carried out in light of the findings of the measurement invariance analysis in this study that legitimizes making intergroup comparisons, because when strict levels of invariance are reached it may be inferred that the differences in performance means are attributable to real variations in measured construct, and not due to measurement biases (Chen et al. 2015; Elosua 2005; Leitgöb et al. 2023).

Consistent with the evidence available in Chile, the comparisons made in the standardization sample showed that although in terms of FSIQ there are no differences in the performance on the WISC-V according to the sex of the participants, a better performance, statistically significant and in favor of the girls is noted on the Processing Speed index (Rosas et al. 2022), a result that was also found in this study and that is consistent with the studies by Kaufman et al. (2016), Hernández et al. (2017), and Grégoire (2020) within other cultural realities. Furthermore, concerning the subtests that comprise the WISC-V, according to the report by Garolera and Navarro (2020) in Chile, girls perform better on tasks that require scanning and visual discrimination, short-term visual memory, and quick and accurate decision-making (Coding and Symbol Search). In contrast, boys are more adept at managing auditory verbal information in working memory (Digit Span), as concluded in the present study. These differences, free of measurement error, could demonstrate that girls process visual stimuli faster or have more precise fine motor skills, better visuomotor coordination, or more effective attentional control than boys. Also, boys show a more remarkable ability to identify auditory verbal information, retain it in temporary storage, and resequence it for problem-solving. It should be noted that these results should be interpreted with caution, given that the effect size of these differences is small, which means that the magnitude of these differences may be limited.

### 4.2. Invariance According to Age Group

As far as the analyses of measurement invariance that considered the age group of the participants (6–8; 9–11; 12–14; 15–16), it should be emphasized that for both the hierarchical five-factor model and the oblique five-factor model the level of configural invariance was satisfied, which endorses the structure of the measurement models tested, i.e., equivalence is noted in the factorial structure (of manifest variables and latent factors) in all the age ranges. This result establishes that the ten primary subtests of the WISC-V are grouped in the same five latent factors in the different age groups of the Chileans evaluated, as in other cultural contexts where this has been explored (Chen et al. 2020; Dombrowski et al. 2021; Reynolds and Keith 2017). 

Although the configuration of the models tested is equivalent among the compared groups, on the following level (metric invariance), it was observed that it is only possible to establish a partial equivalence as to the relevance of each of the subtests on the factor they define when the age of those evaluated is considered, noting a variant functioning particularly in the Matrix Reasoning and Figure Weights subtests, that make up the Fluid Reasoning index on the WISC-V and assess the skills of quantitative and inductive reasoning, simultaneous processing, and abstract thought. Although the little evidence on the matter is diverse, it should be highlighted that Dombrowski et al. (2021) partially achieved a higher level of (scalar) invariance than in the present study. The scalar and residual invariance levels were not reached in the Chilean sample, unlike what was reported by Chen et al. (2020) and Reynolds and Keith (2017) who found levels of strict invariance between the compared groups when their age group of reference was included.

As previously mentioned, their analysis detected imbalances in the subtests of the Fluid Reasoning index (Matrix Reasoning and Figure Weights). In these subtests, the task is for children to discover logical patterns underlying problems presented to them through visual stimuli. Although one might indeed think that in this non-verbal reasoning task it is less likely to find biases, as stated by Wicherts (2016), even tests considered “culture-free” may exhibit measurement biases therefore, according to these results, the possible presence of biased elements of the instrument must be explored. Wicherts (2016) points out that the factors that generate measurement bias in a test can be related to the form of administration, cultural aspects, problems of familiarity with the task, or educational differences, so it would be convenient that new research explores these aspects.

On the other hand, it should be noted that according to the contributions of the CHC theory, the subtests in which imbalances were found measure Fluid Reasoning (*Gf*), i.e., the mental operations used by an individual when faced with a relatively new task that cannot be performed automatically (Flanagan and Dixon 2014). From this definition, resolving new tasks requires the reorganization or transformation of information, perception of relationships between patterns, or inductive and deductive reasoning skills, which develop at a different pace and gradually during childhood and adolescence (Rosas et al. 2019). The results of this study suggest that the items of the Matrix Reasoning and Figure Weight subtests fail to measure these skills in the same way in children and adolescents. The two WISC-V models tested in this study (hierarchical and oblique) place these two subtests in a Fluid Reasoning domain. However, it is worth questioning whether this distribution appropriately accounts for this cognitive domain at all stages of the life cycle. Considering that the results of this study did not demonstrate the equivalence of the measure, in addition to the exploration of bias, it would be advisable to explore other factor models that explore new groupings of the subtests (for example, four-factor models), verify whether there are better factor structures, or different models, within the age ranges studied.

At the professional level, for the Chilean sample, the results of the present study reflect that there are elements affecting the measurement of the skills of quantitative and inductive reasoning through analysis of visual stimuli that must be taken into account in the assessment of cognitive functions with the WISC-V at different stages of development. At the research level, these results do not support the possibility of making performance comparisons on the WISC-V using the age of those evaluated as a comparison variable.

### 4.3. Implications

As previously stated, the evidence found in the present study endorses the use of the WISC-V professionally and in research when the sex of the examinees is considered, since the demonstration of measurement invariance reflects that differences in performance are attributable to real variations in the measured construct and that it is being detected effectively by this instrument. Nevertheless, when the age of those evaluated is considered, the results of this study suggest the need to explore the possible presence of measurement biases more exhaustively.

### 4.4. Future Studies

The differences in performance on the WISC-V in Chilean children indicate that girls are capable of processing visual information, making decisions, and executing them more quickly and efficiently than boys and that boys are better at retention tasks and auditory verbal information processing, which invites further studies to identify cultural, educational, or specific elements of the developmental trajectory of girls that could explain these differences. In this vein, a study that can collect evidence based on the response process would make it possible to approach this objective and enrich the evidence of validity that this instrument currently has in the Chilean population. On the other hand, regarding the variable age group, since the possibility of making intergroup performance comparisons based on this condition needs to be legitimized, it is not recommended that explorations of this type be carried out at the research level. Instead, it is encouraged to carry out studies that allow these findings to be deepened to achieve an understanding of what factors explain the variant functioning in the measurements of Fluid Reasoning that the WISC-V provides, for example, through differential item functioning (DIF).

## Figures and Tables

**Table 1:** Invariance studies with WISC-V according to sociodemographic variables.

Authors	Country	Groups	Results
Chen et al. (2015)	USA	Gender (male female) *n* = 2200	Configural, metric, scalar and residual invariance between sexes with a second-order hierarchical pentafactorial model.
Scheiber (2016)	USA	Ethnicity and gender (African-American, Hispanic, and Caucasian) *n* = 2637	Configural, metric, and scalar invariance for the six groups according to ethnicity and sex with a second-order hierarchical pentafactorial model.
Reynolds and Keith (2017)	USA	Age group (11 separate age groups between 6 and 16 years) *n* = 2200	Configural, metric, scalar, and residual invariance by age group with first-order, hierarchical, and bifactorial factorial models.
Pauls et al. (2020)	Germany	Gender (male female) *n* = 1411	Configural, metric and partial scalar invariance according to sex, with a second-order hierarchical pentafactorial model. Inequality in the Information, Figure Weights, Coding and Cancellation subtests
Smith and Graves (2021)	USA	Gender (boys girls) among African Americans *n* = 647	Configural, metric and partial scalar invariance between sexes, with an oblique model of five first-order factors. Inequality in the Similarities and Coding subtests.
Chen et al. (2020)	Taiwan	Gender (boys girls) and age group (6–8, 9–11, 12–14, and 15–16) *n* = 1034	Configural, metric, scalar, and residual invariance between sexes and age group with a second-order hierarchical pentafactorial model.
Dombrowski et al. (2021)	USA	Sex (male female), age group (6–8, 9–11, 12–14, and 15–16), and clinical diagnosis (ADHD, anxiety, and encephalopathy) *n* = 5359	Configural, metric, and full scalar invariance for sex and clinical diagnosis groups. Partial scalar invariance for the age group. Invariance was tested in an oblique model of five first-order factors. Inequality in the Fluid Reasoning subtests for the age group.
Rodríguez-Cancino et al. (2021)	Chile	Origin (urban-rural) *n* = 480	Configural invariance and partial metric according to origin with a second-order hierarchical pentafactorial model. Inequality in the Similarities subtest.

*Note*: Table created by the authors; regarding gender and sex in Table 1, we present the same labels that authors used (male/female or boys/girls) in their respective studies.

**Table 2:** Distribution of the sample according to sex and age group.

Age Group (in Years)	Boys		Girls		Missing		Total Sample	
	*n*	%	*n*	%	*n*	%	*n*	%
6–8	100	51.5%	94	48.5%	0	0.0%	194	26.2%
9–11	111	47.0%	123	52.1%	2	0.8%	236	31.9%
12–14	94	48.7%	98	50.8%	1	0.5%	193	26.1%
15–16	53	45.3%	64	54.7%	0	0.0%	117	15.8%
Total sex	358	48.3%	379	51.2%	3	0.5%	740	100%

*Note*: Table created by the authors.

**Table 3:** Distribution of subtests according to cognitive domain and reliability coefficients (internal consistency) for the Chilean version of the WISC-V.

Type of Subtest	Cognitive Domain				
	Verbal Comprehension (α = 0.943)	Visual Spatial (α = 0.912)	Fluid Reasoning (α = 0.945)	Working Memory (α = 0.933)	Processing Speed (α = 0.900)
Primary subtest	Similarities (SI; α = 0.921)	Block Design (BD; α = 0.824)	Matrix Reasoning (MR; α = 0.900)	Digit Span (DS; α = 0.907)	Coding (CD; α = 0.898)
	Vocabulary (VC; α = 0.888)	Visual Puzzles (VP; α = 0.903)	Figure Weights (FW; α = 0.941)	Picture Span (PS; α = 0.891)	Symbol Search (SS; α = 0.822)
Complementary subtest	Information (IN; α = 0.910)		Arithmetic (AR; α = 0.900)	Letter-Number Sequencing (LN; α = 0.895)	Cancellation (CA; α = 0.645)
	Comprehension (CO; α = 0.876)				

*Note*: Table created by the authors; α: Cronbach’s alpha.

**Table 4:** WISC-V descriptive statistics by sex and MANOVA results.

	Boys				Girls				Differences		
Subtest/ Index	*M*	*SD*	*Skew.*	*Kurt.*	*M*	*SD*	*Skew.*	*Kurt.*	*F*	*p*	*η* ^2^ *_p_*
BD	10.25	2.919	−0.025	−0.015	10.01	2.861	−0.272	0.202	1.596	0.207	0.002
SI	10.12	2.867	−0.038	0.033	10.09	2.929	−0.175	−0.027	0.013	0.908	<0.001
MR	10.03	2.978	0.409	0.191	10.24	2.786	0.141	−0.265	0.583	0.445	0.001
**DS**	**10.38**	**2.844**	**0.238**	**0.470**	**10.01**	**2.822**	**0.318**	**0.078**	**4.495**	**0.034**	**0.006**
**CD**	**9.75**	**2.765**	**0.480**	**0.187**	**10.72**	**2.655**	**0.167**	**0.422**	**21.994**	**<.001**	**0.030**
VC	10.19	2.890	0.136	−0.520	10.07	2.981	−0.125	−0.624	0.288	0.592	<0.001
FW	10.20	2.965	−0.427	−0.376	10.02	2.954	−0.099	−0.311	0.981	0.322	0.001
VP	10.04	2.955	0.222	−0.366	10.20	2.823	0.155	−0.036	0.09	0.765	<0.001
PS	10.13	3.023	−0.085	−0.146	10.23	2.820	0.155	−0.219	0.26	0.610	<0.001
**SS**	**9.85**	**2.898**	**0.424**	**0.055**	**10.42**	**2.926**	**0.047**	**−0.329**	**6.929**	**0.009**	**0.010**
VCI	100.85	14.048	0.066	−0.303	100.42	14.514	−0.083	−0.625	0.131	.718	<0.001
VSI	100.84	14.411	0.070	−0.423	100.55	13.879	0.072	0.064	0.358	.550	0.001
FRI	100.64	14.194	0.044	−0.567	100.71	14.233	0.107	−0.231	0.033	.855	<0.001
WMI	100.82	13.874	0.223	−0.080	100.05	14.064	0.277	−0.176	0.834	.362	0.001
**PSI**	**98.61**	**13.706**	**0.598**	**0.471**	**103.12**	**13.520**	**−0.110**	**−0.263**	**19.244**	**<.001**	**0.026**
FSIQ	100.78	13.491	0.141	−0.084	100.87	13.934	−0.045	−0.380	0.008	0.930	<0.001

*Note*: *M* = mean; *SD* = standard deviation; *η^2^_p_* = partial eta squared; significant effects in bold.

**Table 5:** Baseline CFA models results.

		Model Fit Indexes						
Group	*χ* ^2^	*df*	*p*	*CFI*	*TLI*	*RMSEA* *[90% CI]*	*SRMR*	*AIC*
**Hierarchical Model**								
Full sample *n* = 740	49.498	30	0.014	0.990	0.985	0.030 [0.013, 0.044]	0.022	34,654.667
Boys *n* = 358	49.982	30	0.012	0.977	0.966	0.043 [0.020, 0.064]	0.032	16,887.609
Girls *n* = 379	32.019	30	0.366	0.998	0.997	0.013 [0.000, 0.042]	0.025	17,626.372
Age 6–8 *n* =194	41.552	30	0.078	0.974	0.961	0.045 [0.000, 0.075]	0.037	9171.730
Age 9–11 *n* = 236	34.542	30	0.259	0.994	0.991	0.025 [0.000, 0.057]	0.030	10,918.633
Age 12–14 *n* = 193	32.072	30	0.413	0.998	0.997	0.013 [0.000, 0.056]	0.034	8942.556
Age 15–16 *n* = 117	39.451	30	0.115	0.975	0.963	0.052 [0.000, 0.092]	0.046	5523.953
**Oblique Model**								
Full sample *n* = 740	28.263	25	0.296	0.998	0.997	0.013 [0.000, 0.033]	0.015	34,643.432
Boys *n* = 358	35.190	25	0.085	0.988	0.979	0.034 [0.000, 0.058]	0.025	16,882.817
Girls *n* = 379	23.328	25	0.558	1.000	1.000	0.000 [0.000, 0.038]	0.020	17,627.681
Age 6–8 *n* = 194	23.448	25	0.551	1.000	1.000	0.000 [0.000, 0.053]	0.029	9163.626
Age 9–11 *n* = 236	26.327	25	0.390	0.998	0.997	0.015 [0.000, 0.055]	0.026	10,920.418
Age 12–14 *n* = 193	22.375	25	0.614	1.000	1.000	0.000 [0.000, 0.050]	0.023	8944.859
Age 15–16 *n* = 117	34.680	25	0.094	0.975	0.955	0.058 [0.000, 0.100]	0.041	5529.182

*Note: χ*^2^ = Chi-square; *df* = degrees of freedom; *CFI* = comparative fit index; *TLI* = Tucker Lewis index; *RMSEA* = root mean square error of approximation; *SRMR* = standardized residual root mean square; *AIC* = Akaike Information Criterion.

**Table 6:** WISC-V hierarchical multigroup CFA fit indexes and the comparison of invariance models by sex.

Invariance Model	Model Fit Indexes								Model Comparison					
	*χ* ^2^	*df*	*p*	*CFI*	*TLI*	*RMSEA* *[90% CI]*	*SRMR*	*AIC*	*Comparison*	Δ*χ^2^*	Δ*df*	Δ*CFI*	Δ*RMSEA*	Δ*SRMR*
M_0_: Configural	81.584	60	0.033	0.988	0.982	0.031 [0.009, 0.047]	0.028	34,513.980	-	-	-	-	-	-
M_1_: Metric (First-Order Loadings)	87.433	65	0.033	0.988	0.983	0.031 [0.009, 0.046]	0.034	34,509.755	M_1_–M_0_	5.849	5	0.000	0.000	0.006
M_2_: Metric (Second-Order Loadings)	87.680	69	0.064	0.990	0.987	0.027 [0.000, 0.043]	0.035	34,502.287	M_2_–M_1_	0.247	4	0.002	−0.004	0.001
M_3_: Scalar (Intercepts of the Indicators)	101.126	74	0.019	0.985	0.982	0.032 [0.013, 0.046]	0.039	34,505.703	M_3_–M_2_	13.446	5	−0.005	0.005	0.004
M_4_: Scalar (Intercepts of the First-Order Factors)	127.401	78	<0.001	0.973	0.969	0.041 [0.028, 0.054]	0.046	34,524.338	M_4_–M_3_	26.275	4	−0.012	0.009	0.007
M_5_: Residual (Disturbances of First-Order Factors)	155.410	87	<0.001	0.963	0.962	0.046 [0.034, 0.058]	0.054	34,534.381	M_5_–M_4_	28.009	9	−0.010	0.005	0.008
M_6_: Residual (Uniqueness of the Indicators)	171.796	97	<0.001	0.959	0.962	0.046 [0.034, 0.057]	0.064	34,533.061	M_6_–M_5_	16.386	10	−0.004	0.000	0.010

*Note: χ*^2^ = Chi-square; *df* = degrees of freedom; *CFI* = comparative fit index; *TLI* = Tucker Lewis index; *RMSEA* = root mean square error of approximation; *SRMR* = standardized residual root mean square; *AIC* = Akaike Information Criterion.

**Table 7:** WISC-V hierarchical multigroup CFA fit indexes and comparison of invariance models by age group.

Invariance Model	Model Fit Indexes								Model Comparison					
	*χ* ^2^	*df*	*p*	*CFI*	*TLI*	*RMSEA* *[90% CI]*	*SRMR*	*AIC*	*Comparison*	Δ*χ*^2^	Δ*df*	Δ*CFI*	Δ*RMSEA*	Δ*SRMR*
M_0_: Configural	148.313	121	0.046	0.986	0.979	0.035 [0.005, 0.053]	0.036	34,556.882	-	-	-	-	-	-
M_1_: Metric (First-Order Loadings)	210.727	136	<0.001	0.961	0.949	0.054 [0.040, 0.068]	0.072	34,587.547	M_1_–M_0_	62.414	15	−0.025	0.019	0.036
M_1a_: Metric–Partial (First-Order Loadings)	181.533	132	0.002	0.974	0.965	0.045 [0.027, 0.060]	0.057	34,568.112	M_1a_–M_0_	33.220	11	−0.012	0.01	0.021
M_2_: Metric–Partial (Second-Order Loadings)	286.790	163	<0.001	0.936	0.929	0.064 [0.052, 0.076]	0.094	34,607.742	M_2_–M_1_	105.257	31	−0.038	0.019	0.037
M_3_: Scalar–Partial (Intercepts of the Indicators)	321.900	175	<0.001	0.924	0.922	0.067 [0.056, 0.079]	0.094	34,616.754	M_3_–M_2_	35.110	12	−0.012	0.003	0.000
M_4_: Scalar–Partial (Intercepts of the First-Order Factors)	337.396	184	<0.001	0.921	0.923	0.067 [0.056, 0.078]	0.094	34,616.174	M_4_–M_3_	15.496	9	−0.003	0.000	0.000
M_5_: Residual–Partial (Disturbances of First-Order Factors)	292.425	177	<0.001	0.940	0.939	0.059 [ 0.047, 0.071]	0.089	34,588.607	M_5_–M_4_	−44.971	−7	0.019	−0.008	−0.005
M_6_: Residual–Partial (Uniqueness of the Indicators)	397.257	204	<0.001	0.900	0.912	0.072 [0.061, 0.082]	0.144	34,644.369	M_6_—M_5_	104.832	27	−0.040	0.013	0.055

*Note: χ*^2^ = Chi-square; *df* = degrees of freedom; *CFI* = comparative fit index; *TLI* = Tucker Lewis index; *RMSEA* = root mean square error of approximation; *SRMR* = standardized residual root mean square; *AIC* = Akaike Information Criterion.

**Table 8:** Fit indexes of the multigroup CFA in the WISC-V oblique model and comparison of the invariance models according to sex.

Invariance Model	Model Fit Indexes								Model Comparison					
	*χ* ^2^	*df*	*p*	*CFI*	*TLI*	*RMSEA* *[90% CI]*	*SRMR*	*AIC*	*Comparison*	Δ*χ*^2^	Δ*df*	Δ*CFI*	Δ*RMSEA*	Δ*SRMR*
M_0_: Configural	58.518	50	0.191	0.996	0.992	0.022 [0.000, 0.042]	0.022	34,510.498	-	-	-	-	-	-
M_1_: Metric (Loadings)	61.988	55	0.241	0.996	0.994	0.019 [0.000, 0.039]	0.028	34,503.967	M_1_–M_0_	3.470	5	0.000	−0.003	0.006
M_2_: Scalar (Intercepts of the Indicators)	74.659	60	0.096	0.992	0.989	0.026 [0.000, 0.043]	0.033	34,506.639	M_2_–M_1_	12.671	5	−0.004	0.007	0.005
M_3_: Residual (Disturbances of First-Order Factors)	94.906	70	0.026	0.987	0.983	0.031 [0.011, 0.046]	0.046	34,506.886	M_3_–M_2_	20.247	10	−0.005	0.005	0.013

*Note: χ*^2^ = Chi-square; *df* = degrees of freedom; *CFI* = comparative fit index; *TLI* = Tucker Lewis index; *RMSEA* = root mean square error of approximation; *SRMR* = standardized residual root mean square; *AIC* = Akaike Information Criterion.

**Table 9:** Fit indexes of the multigroup CFA in the WISC-V oblique model and comparison of the invariance models according to age group.

Invariance Model	Model Fit Indexes								Model Comparison					
	*χ^2^*	*df*	*p*	*CFI*	*TLI*	*RMSEA* *[90% CI]*	*SRMR*	*AIC*	*Comparison*	Δ*χ*^2^	Δ*df*	Δ*CFI*	Δ*RMSEA*	Δ*SRMR*
M_0_: Configural	108.071	100	0.273	0.996	0.992	0.021 [0.000, 0.045]	0.029	34558.084	-	-	-	-	-	-
M_1_: Metric (Loadings)	161.543	111	0.001	0.974	0.958	0.050 [0.032, 0.066]	0.064	34589.475	M_1_–M_0_	53.472	11	−0.022	0.029	0.035
M_1a_: Metric–Partial (Intercepts of th Indicators)	166.947	112	<0.001	0.972	0.954	0.051 [0.034, 0.067]	0.067	34592.216	M_1a_–M_0_	58.876	12	−0.020	0.030	0.038
M_2_: Scalar (Intercepts of the Indicators)	190.807	124	<0.001	0.966	0.950	0.054 [0.038, 0.069]	0.066	34592.167	M_2–_M_1a_	23.860	12	−0.006	0.003	−0.001
M_3_: Residual (Disturbances of First-Order Factors)	271.746	151	<0.001	0.938	0.926	0.066 [0.053, 0.078]	0.102	34624.586	M_3_–M_2_	80.939	27	−0.028	0.011	0.036

*Note*: *χ^2^* = Chi-square; *df* = degrees of freedom; *CFI* = comparative fit index; *TLI* = Tucker Lewis index; *RMSEA* = root mean square error of approximation; *SRMR* = standardized residual root mean square; *AIC* = Akaike Information Criterion.

**Table 10:** Summary of the reached levels of measurement invariance analyses according to sex and age group.

	Sex		Age Group	
Level	Hierarchical	Oblique	Hierarchical	Oblique
Configural	Yes	Yes	Yes	Yes
Metric	Yes	Yes	Partial	Partial
Scalar	Yes	Yes	No	No
Residual	Yes	Yes	No	No

*Note*: Table created by the authors; “Yes” means that a certain level was reached.

## Data Availability

Data are not available due to ethical or privacy restrictions.
